# 4-Phenylbutyrate Plus Wildtype GAT-1 Augmentation: A Dual Therapy to Rescue SLC6A1 Variant-Associated Developmental and Epileptic Encephalopathy

**DOI:** 10.3390/genes17080983

**Published:** 2026-08-21

**Authors:** Aiden James Delahanty, Kaitlin James, Emma Grace Carter, Ziang Debbie Song, Juexin Wang, Melissa Bassette, Jing-Qiong Kang

**Affiliations:** 1 Department of Neurology, Vanderbilt University Medical Center, Nashville, TN 37232, USA; aidelahanty@g.ucla.edu (A.J.D.); ziang.song.1@vumc.org (Z.D.S.); melissa.bassett@vumc.org (M.B.); 2 Department of Pediatrics, Vanderbilt University Medical Center, Nashville, TN 37232, USA; kaitlin.c.james@vumc.org (K.J.); emma.g.carter@gmail.com (E.G.C.); 3 Department of Biomedical Engineering and Informatics, Luddy School of Informatics, Computing, and Engineering, Indiana University, Indianapolis, IN 46202, USA; wangjuex@iu.edu; 4 Department of Pharmacology, Vanderbilt University, Nashville, TN 37232, USA; 5 Vanderbilt Kennedy Center for Research on Human Development, Vanderbilt University, Nashville, TN 37232, USA

**Keywords:** SLC6A1, GABA transporter 1 (GAT-1), developmental and epileptic encephalopathy (DEE), 4-phenylbutyrate (PBA), pharmacochaperone, endoplasmic reticulum (ER), gene therapy

## Abstract

Background: Pathogenic variants in SLC6A1, which encodes the γ-aminobutyric acid (GABA) transporter GAT-1, cause developmental and epileptic encephalopathies (DEEs) through reduced GABA uptake, impaired transporter trafficking, and functional haploinsufficiency. 4-phenylbutyrate (PBA) is a clinically available small molecule with chemical-chaperone and histone-deacetylase-inhibitor activities that can rescue misfolded GABAergic proteins, but variant-level rescue data are needed to guide precision treatment. Methods: We report a novel de novo missense mutation p.Ala305Val in GAT-1 encoding SLC6A1, in a patient with myoclonic-atonic epilepsy and a developmental and epileptic encephalopathy phenotype. Ala305Val was compared with the residue-matched comparator p.Ala305Thr (Ala305Thr). Variant effects were evaluated by (i) protein-structure prediction across nine stability-prediction algorithms using the cryo-EM-derived human GAT-1 template (PDB 7Y7W); (ii) 3H-GABA uptake assays in HEK293T cells and in human iPSC-derived astrocytes and cortical neurons; (iii) live-cell confocal microscopy of ER colocalization; (iv) pharmacologic rescue with PBA, TUDCA and salubrinal (v) and GAT-1 cDNA gene-augmentation, alone and in combination with PBA. Results: AI-based stability predictors uniformly indicated destabilization of GAT-1 p.Ala305Val and GAT-1 p.Ala305Thr. GAT-1 p.Ala305Val reduced 3H GABA uptake across HEK293Ts, astrocytes, and neurons. The mutant transporter accumulated within the endoplasmic reticulum (ER), with ER colocalization rising from approximately 30% in wildtype to ~80% in GAT-1 p.Ala305Val; PBA reduced ER retention to approximately ~40% and restored total GAT-1 fluorescence toward wildtype levels. Pharmacochaperones (PBA, TUDCA) restored GABA uptake for the mutant transporters. Wildtype GAT-1 gene augmentation improved GABA uptake in the heterozygous condition but combined PBA plus wildtype allele augmentation produced rescue greater than either intervention alone in the available dose-response ranges. Conclusions: GAT-1 p.Ala305Val is a trafficking-impaired, loss-of-function variant whose dysfunction is amenable to two convergent therapeutic axes: pharmacologic correction of folding and trafficking, and augmentation of functional transporter expression. These findings support a two-pronged precision-medicine framework for SLC6A1-related DEEs in which PBA increased the transporter function augmented by genetic approaches.

## 1. Significance of the Study

This work links the patient-derived SLC6A1 p.Ala305Val variant to a defined molecular mechanism; briefly, the GAT-1 destabilization, ER retention, and reduced GABA uptake demonstrates that the deficit is reversible by two independent interventions that converge at the same downstream endpoint of a functional surface GAT-1 transporter. Because PBA is already clinically deployable for pediatric use and GAT-1 cDNA augmentation models a future viral or non-viral gene therapy to upregulate the wildtype GAT-1, the combined rescue approach provides a feasible path for precision medicine in SLC6A1-related DEEs: pharmacochaperoning corrects the folding impairment and remodels the ER folding environment while wildtype GAT-1 augmentation increases the functional GAT-1 pool available for rescue.

## 2. Introduction

γ-Aminobutyric acid (GABA) transporter 1 (GAT-1), encoded by SLC6A1, is the principal neuronal and astrocytic transporter that clears GABA from the synaptic cleft and extrasynaptic compartments in the mammalian central nervous system [1,2]. By terminating GABAergic neurotransmission and shaping tonic inhibition, GAT-1 helps maintain the excitation–inhibition balance whose loss underlies developmental and epileptic encephalopathies (DEEs). Pathogenic SLC6A1 variants disrupt this homeostasis and cause a spectrum of DEEs typically characterized by myoclonic-atonic seizures, absence seizures, atypical absences, global developmental delay, autism-spectrum features, attention-deficit/hyperactivity symptoms, and sleep or behavioral disturbances [3,4,5].

We have previously characterized the mutant GAT-1 function and trafficking in various cell and mouse models [6,7]. Work from our laboratory and others has shown that the majority of pathogenic SLC6A1 missense variants do not simply abolish substrate translocation. Rather, they destabilize the folded transporter, lead to glycosylation arrest and endoplasmic reticulum (ER) retention, and produce a functional haploinsufficiency in which mutant protein fails to reach the plasma membrane in a transport-competent state [6,8,9,10]. This trafficking-linked loss of function rather than a pure transporter deficiency reframes SLC6A1-related DEEs. This also suggests that SLC6A1-related DEEs are in part, a proteostasis disease and points toward therapeutic strategies that target folding and forward trafficking rather than channel pharmacology alone.

4-phenylbutyrate (PBA), an FDA-approved small molecule used clinically for urea-cycle disorders, has chemical chaperone activity, low-grade HDAC-inhibitory activity, and ER-stress-modulating activity. Importantly, we have identified PBA, which can restore the GABA uptake and mitigate seizures in the cell and mouse models [7,11] as well as human patients bearing the SLC6A1 variants [12,13] and GABRA1 variants [14]. In preclinical SLC6A1 and GABA_A_-receptor models, PBA improves protein folding, reduces ER stress, increases surface expression, restores GABA uptake or GABA_A_ receptor function, and mitigates seizure phenotypes [7,11]. The phase I clinical trial of glycerol phenylbutyrate (Ravicti) in SLC6A1 and STXBP1 (NCT04937062) provides a strong translational foothold for PBA in rare genetic epilepsy especially for SLC6A1 variants. Although it is still at an observational stage, the feedback from patients is promising. However, clinical observation and preclinical work indicate that the magnitude and mechanism of PBA rescue are variant-dependent, suggesting variant-based study may provide better precision for treatment development.

We incorporated AI tools for mutant protein stability prediction. Computational protein stability prediction is increasingly used to triage variants of uncertain significance and to forecast the structural consequences of missense substitutions [15,16]. We have integrated the AI prediction with experimental validation to evaluate the impact of SLC6A1 [9] and GABRA1 variants [14]. For a transporter such as GAT-1, structural data are now available at near-atomic resolution [17].

In silico modeling predicts whether a specific residue substitution is expected to impair protein folding or trafficking, and these predictions are experimentally testable using uptake and biochemical assays.

Here we report the characterization of impaired trafficking and functional rescue of SLC6A1 p.Ala305Val, a newly identified de novo missense variant associated with DEE. We have previously elucidated the mechanism of pharmacochaperoning and HDAC inhibition as well as the effect of genetic upregulation of the folding machinery like Bip and Calnexin on GAT-1 variants [18]. The central translational hypothesis is that pharmacochaperoning is the major mechanism underlying GAT-1 upregulation for SLC6A1 variants. Here we evaluate the convergent therapeutic axes: correction of protein misfolding with a pharmacochaperone (PC) with PBA, TUDCA and salubrinal, and augmentation of the wildtype allele with or without the treatment of PBA. Both PBA [19], known as chemical chaperone and TUDCA [20], a hydrophilic bile salt, can enhance protein folding and are approved by FDA to treat urea cycle disorders. Considering the ongoing gene therapy as a promising treatment option for many DEEs and epilepsy, the study provides a proof of concept understanding for two different approaches, small molecules and gene therapy, as complementary rather than redundant for trafficking-impaired SLC6A1 variants. PBA can enhance the efficacy of gene therapy by maximizing the functional contribution of both the wildtype allele and rescuable mutant allele, while promoting the efficient clearance of misfolded protein.

## 3. Methods

### 3.1. Patient Ascertainment, Clinical Evaluation, and Genetic Diagnosis

The proband and available family members were evaluated at Vanderbilt University Medical Center. Written informed consent for the use of clinical and genetic information was obtained from the parent(s) prior to inclusion. Abstracted clinical data included age of seizure onset, seizure semiology and frequency, developmental milestones, antiseizure medication exposure and response, video-EEG findings, and neurodevelopmental milestones and comorbidities such as autism-spectrum disorder and attention-deficit/hyperactivity symptoms were assessed using standard instruments. Saliva samples were submitted for CLIA-approved clinical genetic testing on the Invitae Comprehensive Epilepsy Gene Panel (Invitae, San Francisco, CA, USA). The SLC6A1 p.Ala305Val variant was identified de novo.

### 3.2. Protein-Structure Prediction and Machine-Learning Analyses

Stability and flexibility changes induced by GAT-1 p.Ala305Val and p.Ala305Thr were predicted from the cryo-EM-derived human GAT-1 template (PDB 7Y7W) [17], which was selected for its 2.9-Å resolution and complete coverage of the GAT-1 p.Ala305 site. Point mutations were introduced in silico, and ΔΔG predictions were obtained from DynaMut2 [16], mCSM [15], SDM [21], DUET [15], I-Mutant, MUpro (SVM and neural-network variants), INPS-MD [22], and MAESTROweb [23]. The same structural template was used across models for consistency, and effects were interpreted in the context of side-chain polarity, hydrophobic packing, steric fit, and transmembrane architecture. Negative ΔΔG values indicate predicted destabilization.

### 3.3. Expression Constructs and Site-Directed Mutagenesis

Plasmids encoding rat GAT-1 tagged with enhanced yellow fluorescent protein (EYFP) were constructed in the pCMV mammalian expression vector as previously described [8,9]. The GAT-1 p.Ala305Val and p.Ala305Thr missense substitutions were introduced into the wildtype GAT-1 coding sequence using the QuikChange Site-Directed Mutagenesis Kit (Agilent, Santa Clara, CA, USA, Cat No. 200515), transformed into DH5α competent cells, plated under antibiotic selection, and verified by Sanger sequencing of single colonies. Endotoxin-controlled plasmid stocks were prepared with the QIAGEN Maxiprep kit (QIAGEN, Germantown, MD, USA, Cat. NO. 12165X4) and stored at −20 °C. For heterozygous condition modeling, wildtype and mutant constructs were co-expressed at equal mass ratios; for homozygous or mutant-only condition, mutant cDNA alone was transfected.

### 3.4. Polyethylenimine (PEI) Transfection

Standard transfection protocols were performed using human embryonic kidney 293T (HEK293T) cells [24]. 24 h before transfection HEK293T cells were split equally into plates. For radiolabeling GABA uptake, 1 µg of the cDNAs with PEI at a ratio of 1:2.5 µL was transfected in 35 mm dish or in 60 mm dish for Western blot. The cDNAs were combined with Dulbecco’s Modified Eagle’s Medium (DMEM) and a PEI/DMEM mixture. For total protein expression, 3 µg cDNAs were used for transfection. Transfected HEK293T cells were incubated for 48 h. After incubation, proteins were harvested as described below.

## 4. Cell Culture, iPSC Differentiation for Neurons and Astrocytes, and Transfection

HEK293T cells were maintained in Dulbecco’s Modified Eagle’s Medium (DMEM) with 10% fetal bovine serum and 1% penicillin/streptomycin. Transfections used polyethylenimine (PEI) at a 1:2.5 mass ratio of cDNA to PEI, with 1 μg cDNA per 35 mm dish for GABA uptake assays and 3 μg cDNA per dish for total-protein analysis. Transfections were incubated for 48 h prior to assay. Human induced pluripotent stem cells (iPSCs; Thermo Fisher A18945) (Thermo Fisher, Waltham, MA, USA) were maintained and differentiated into neural progenitor cells (NPCs), astrocytes, and cortical inhibitory neurons following our previously validated protocol [6]. NPC induction used the STEMdiff SMADi Neural Induction Kit (STEMCELL Technologies, Vancouver, BC, Canada). Astrocytes were previously differentiated from P1 NPCs in Astrocyte Medium (ScienCell, Carlsbad, CA, USA) for 25–30 days; medium was refreshed every other day, and cells were passaged at ≈70% confluence. We here modified the astrocyte differentiation protocol with Stemdiff Astrocyte Differentiation Medium for about 30 days and then with Stemdiff Astrocyte mature medium. Astrocyte identity was confirmed by S100β and glial fibrillary acidic protein (GFAP) immunostaining, with >95% of cells adopting astrocytic morphology and ≈80% staining GFAP-positive, consistent with prior reports. Cortical inhibitory neurons were differentiated from passage two NPCs at day 10 of induction, harvested at differentiation days (60–65), and validated by NeuN, DLX, synapsin, and synaptophysin immunostaining. Astrocytes and neurons were transfected with 1 μg cDNA per 35 mm dish using Lipofectamine.

### 4.1. ^3^H-GABA Uptake Assay

Transporter function was measured using a modified ^3^H GABA uptake assay [8,25]. HEK293T cells were seeded at 1.5 × 10^5^ cells per 35 mm dish three days before assay and transfected with 1 μg of wildtype or mutant GAT-1 cDNA at 24 h after seeding. Uptake was performed 48 h after transfection. Cells were preincubated with uptake buffer for 15 min, then exposed to 1 μCi/mL ^3^H GABA and 10 μM unlabeled GABA at room temperature for 30 min. After washing, cells were lysed with 0.25 N NaOH for 1 h, neutralized with glacial acetic acid, and counted on a liquid-scintillation counter (QuantaSmart). GABA flux (pmol/μg protein/min) was averaged from technical triplicates per condition; untransfected wells served as background and were subtracted. Mutant uptake was normalized to wildtype within each experiment, with wildtype defined as 100%. The GAT-1-selective inhibitor Cl-966 (100 μM) and the GAT-3-selective inhibitor SNAP5114 (30 μM, astrocytes only) were used as pharmacologic specificity controls.

### 4.2. Pharmacologic and Wildtype GAT-1 Allele Augmentation Rescue

GAT-1 cDNA augmentation was modeled by titrating wildtype GAT-1 plasmid (0.125, 0.25, or 0.5 μg) into mutant-expressing astrocyte cultures while holding total transfected DNA constant with carrier PcDNA. GAT-1 p.Ala305Val (A305V) and GAT-1 Ser295Leu (S295L) were tested in parallel as trafficking-impaired comparator variants. For combined rescue, GAT-1 p.Ala305Val and GAT-1 Ser295Leu expressing cells received fixed GAT-1 augmentation in the presence of increasing PBA concentrations (0.5, 1, or 2 mM, 24 h). TUDCA (100 μM, 24 h) or salubrinal (15 μM, 24 h) was also applied in some experiments. For genetic augmentation of GAT-1 with cDNAs, GAT-1 constructs were co-transfected with empty PcDNA control at desired mass ratios to normalize the total transfected cDNA amount when necessary. The combined approach is described throughout as gene augmentation rather than gene therapy because in vivo viral delivery in animals was not performed in this study.

### 4.3. Live-Cell Confocal Microscopy and ER Colocalization

Cells were plated on poly-D-lysine–coated, glass-bottom dishes at 1–2 × 10^5^ cells/dish and co-transfected with 0.5 μg of wildtype or mutant GAT-1 tagged with enhanced yellow fluorescent protein (EYFP) (GAT-1^YFP^) and 0.5 μg of enhanced yellow fluorescent protein (ECFP)-tagged ER marker (ER^CFP^) with using polyethylenimine (PEI). Live-cell confocal imaging was performed 48 h post-transfection on a Zeiss LSM 510 inverted laser-scanning microscope with a 63×/1.4 NA oil-immersion objective and multi-track excitation at 458 nm (ECFP) and 514 nm (EYFP). Single confocal sections were averaged from eight frames to reduce noise unless otherwise noted. ER colocalization was quantified in MetaMorph and ImageJ as the percentage of GAT-1^YFP^ fluorescence overlapping with the ER marker, and total GAT-1 fluorescence intensity was measured in parallel.

### 4.4. Western Blot of GAT-1 Expression

Transfected cells were washed in PBS, lysed in RIPA buffer (20 mM Tris, 20 mM EGTA, 1 mM DTT, 1 mM benzamidine, 0.01 mM PMSF, 0.005 g/mL leupeptin, 0.005 g/mL pepstatin) for 30 min at 4 °C, and resolved by SDS-PAGE. Membranes were probed with rabbit anti-GAT-1 (Synaptic Systems, Goettingen Germany, Cat No. 274102; 1:200) and quantified with Odyssey. GAT-1 ran as three bands at 108, 96, and 90 kDa, corresponding to mature glycosylated, partially glycosylated, and immature core-glycosylated forms, respectively, consistent with our prior reports [8]. Mature/immature band-intensity ratios were used as a maturation readout of GAT-1.

### 4.5. Statistical Analysis

Numerical data were expressed as mean ± SEM. Proteins were quantified by Odyssey software and data were normalized to loading controls and then to wildtype GAT-1, which was arbitrarily taken as 1 in each experiment. The radioactivity of GABA uptake was measured in a liquid scintillator with QuantaSmart. The flux of GABA (pmol/µg/min) in the wildtype GAT-1 samples was arbitrarily taken as 100% each experiment. The fluorescence intensities from confocal microscopy experiments were determined using MetaMorph imaging software and the measurements were carried out in ImageJ as modified from previous description [2,14]. For statistical significance, we used one-way analysis of variance (ANOVA) with Newman–Keuls test or Student’s unpaired *t*-test. In some cases, one sample *t*-test was performed (GraphPad Prism, La Jolla, CA, USA), and statistical significance was taken as *p* < 0.05.

## 5. Results

### 5.1. A Novel Variant SLC6A1 p.Ala305Val Associated with DEE

**Figure 1:**
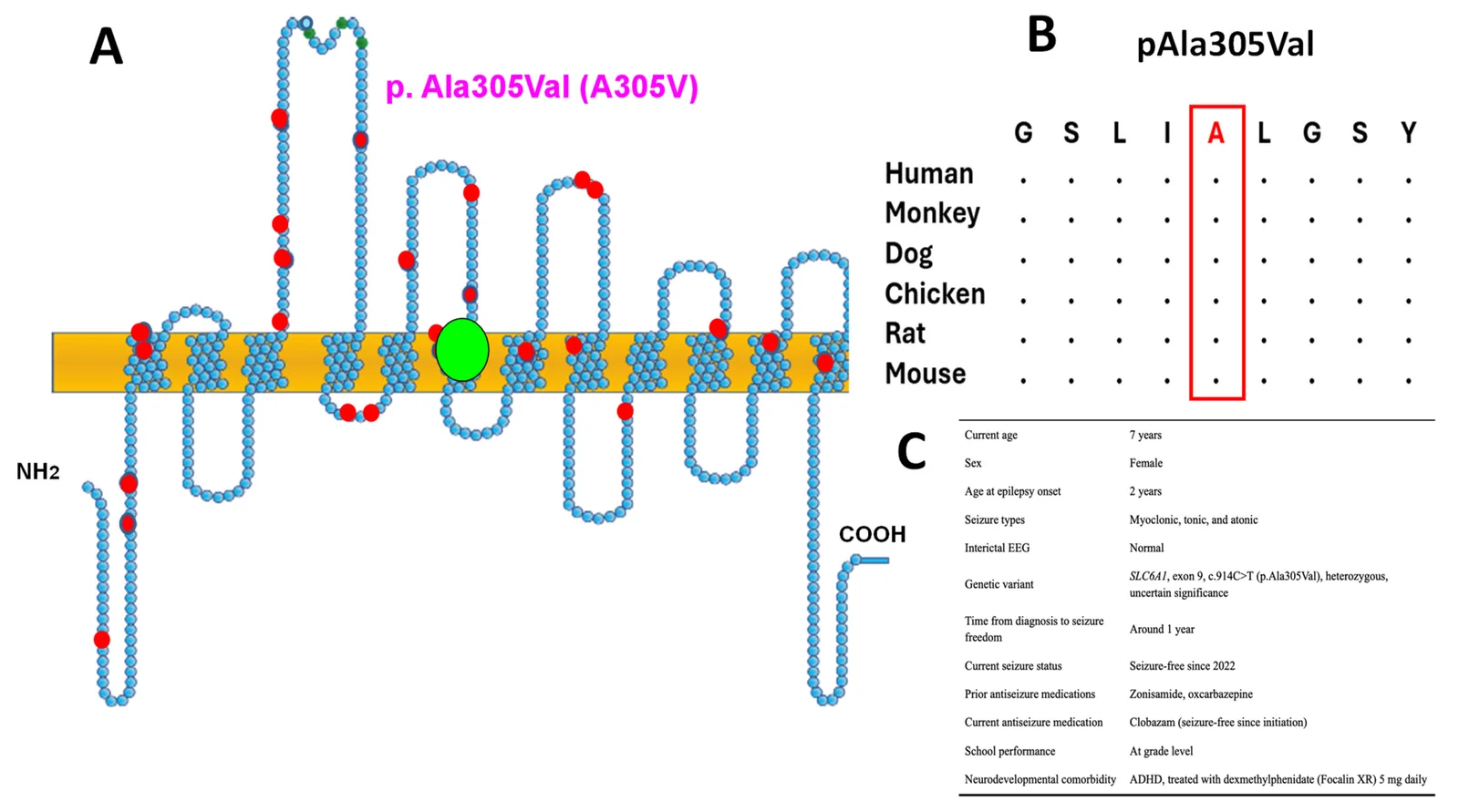
**A De Novo mutation in SLC6A1, resulting in GAT-1 Ala305Val was identified in a patient associated with developmental and epileptic encephalopathies and experimental design of the study.** (**A**) Schematic representation of GAT-1 protein topology and locations of GAT-1 variants previously identified in patients associated with developmental and epileptic encephalopathies (DEEs). It is predicted that GAT-1 contains 12 transmembrane domains. P.Ala305Val is located at the 4th intracellular loop of the GAT-1 protein. The positions of variants are based on the published LeuT crystal structure. We included GAT-1 p.Ala305Thr associated with other DEEs as the reference pedigree and genotype. 914C>T variation resulting in GAT-1 p.Ala305Val was found, de novo, in a two year-old girl but not in the mother and father. (**B**) Amino acid sequence homology shows that Alanine (A) at residue 305V is highly conserved in GAT-1 in humans (Accession NO.NP_003033.3) and across species as shown in red boxed region. (**C**) Clinical information of the patient at 7 years old.

Previous studies have identified mutations in *SLC6A1* associated with a spectrum of epilepsy syndromes and neurodevelopmental disorders [1,2,8,15,16]. We have previously studied *SLC6A1* mutations associated with a wide spectrum of disease phenotypes including MAE, Lennox–Gastaut syndrome, autism, ADHD, and neurodevelopmental delay due to various degrees of loss of GAT-1 function and genetic background of affected individuals. Most previously assayed mutations as denoted with red dots are de novo (Figure 1A). Here we identified 914C to T mutation in *SLC6A1* resulting in a change in a conserved residue Alanine 305 to Valine (A305V) (Figure 1A) in the GAT-1 protein (Figure 1B). This variant of unknown significance was not identified in parents, suggesting de novo. The mutant amino acid of Alanine is conserved across species, suggesting the variation in the amino acid may have a significant impact on the transporter protein conformation and function.

The girl’s early life history was unremarkable until the presentation of seizures. The first seizure onset was at two years old and the seizure type was myoclonic, tonic and atonic. The child was treated with zonisamide, oxcarbazepine and clobazam and achieved seizure-free status since clobazam. The child performs at grade level with treatment for ADHD (Focalin (dexmethylphenidate) 5 mg daily) (Figure 1C).

### 5.2. Computational Stability Prediction Across Nine Algorithms Supports Destabilization at Ala305, Which Was Validated in Cells

**Figure 2:**
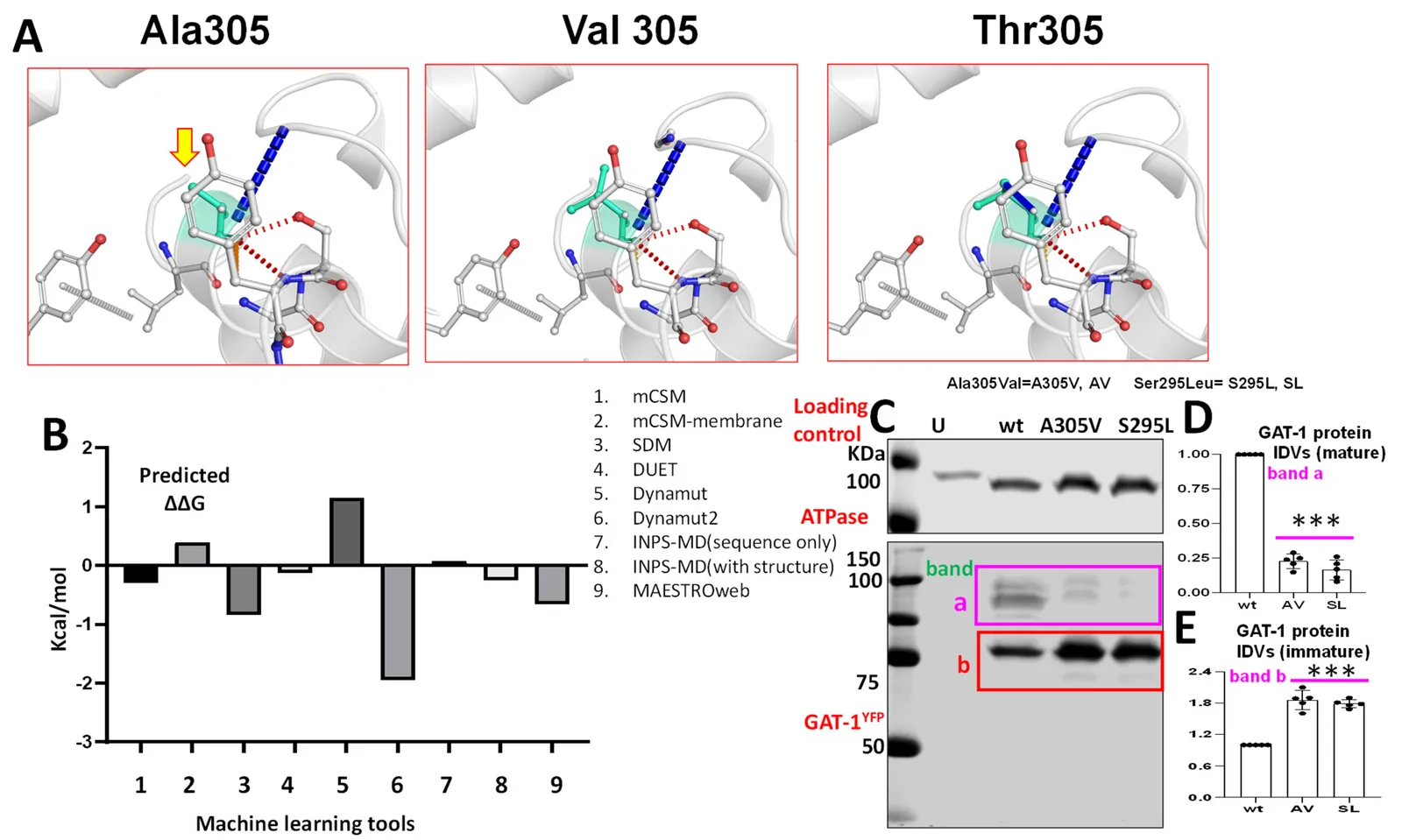
**Both artificial intelligence tools and biochemical assay predict reduced stability of the mutant GAT-1 p.Ala305Val protein.** (**A**) DynaMut2-predicted local environments around residue 305V (yellow arrow) in the wildtype (WT) GAT-1 and the GAT-1 p.Ala305Val (A305V, AV) mutants, using the human GAT-1 crystal structure (PDB: 7Y7W). Hydrophobic interactions are shown in green lines; polar interactions (e.g., hydrogen bonds) are shown in orange dashed lines. (**B**) ΔΔG stability predictions for Ala305Val (**B**) across multiple computational tools. Negative values indicate destabilization. Methods include DynaMut2 (Dyn), mCSM, SDM, DUET, I-Mutant, MUpro (SVM and NN), INPS, and MAESTROweb (MAE). (**C**–**E**). HEK293T cells were transfected with the wildtype GAT-1^HA^ in combination of wildtype GAT-1^YFP^; GAT-1 p.Ala305Val(A305V, AV)^YFP^ or with GAT-1 p. Ser295Leu (S295L, SL)^YFP^ at 1:1 cDNA ratio for 48 h. The total cell lysates were analyzed with SDS-PAGE. U stands for untransfected control. The membrane was immunoblotted with a mouse monoclonal GFP antibody (**C**). The total protein IDVs of the mature GAT-1 (band a, purple boxed) in the purple box (**D**) or the immature GAT-1 (band b, red boxed) in the red box (**E**) were measured. ATPAse was used as loading control. In (**D**,**E**), *** *p* < 0.001 vs. wt, N = 5 gels from 5 different transfections. One-way analysis of variance (ANOVA) and Tukey post hoc test for multiple comparisons.

To establish a structural expectation for the experimental program, p.Ala305Val and p.Ala305Thr were modeled in silico against the 2.9-Å cryo-EM human GAT-1 structure (PDB 7Y7W). DynaMut2 indicated that introducing a polar threonine hydroxyl into a predominantly hydrophobic transmembrane pocket creates polarity mismatch and local steric strain at p.Ala305Thr, while the valine substitution in p.Ala305Val increases hydrophobic bulk and creates steric hindrance in a confined packing environment (Figure 2A). Across the panel of nine stability predictors (DynaMut2, mCSM, SDM, DUET, I-Mutant, MUpro-SVM, MUpro-NN, INPS-MD, MAESTROweb), the direction of the effect favored destabilization for both substitutions, with a mean ΔΔG of approximately −1.4 kcal/mol for p.Ala305Thr and −0.5 kcal/mol for p.Ala305Val; DUET predicted the largest single effect for p.Ala305Val (−3.4 kcal/mol) (Figure 2B). Across most stability prediction tools (Figure 2B), negative ΔΔG values were observed for both Ala305Thr (Supplementary Figure S1) and p.Ala305Val variants, with an average of −1.4 kcal/mol for p.Ala305Thr and −0.5 kcal/mol for p.Ala305Val. While the magnitude of ΔΔG varied between tools, most computational tools predicted destabilization of the mutant protein, with DUET estimating the largest effect for p.Ala305Thr (−3.4 kcal/mol). Only two AI tools (mCSM-membrane and Dynamut) predicted that the p.Ala305Val mutant was not destabilized and Dynamut predicted that the p.Ala305Thr mutant protein was not destabilized. This consistent pattern across algorithms indicates a robust destabilization signal in the mutant GAT-1.

We then used a biochemical assay to determine the mutant protein stability. In the cells expressing the wildtype or the mutant GAT-1^YFP^, the GAT-1 p.Ala305Val protein at the ~108 KDa in the purple boxed region representing the mature forms of GAT-1 (band a) was reduced compared to the wildtype (1 vs. 0.228 ± 0.023) but similar to GAT-1 p.Ser295Leu (0.16 ± 0.032), which has been identified to cause a loss of GABA function due to reduced functional GAT-1 protein at the total and cell surface levels (Figure 2C,D and Supplementary Figure S2). By contrast, the lower band (band b) at ~90 KDa representing the immature form in the red boxed region was higher in the mutant GAT-1 p.Ala305Val (1.858 ±0.08) and GAT-1 p.Ser295Leu (1.785±0.0357) compared with the wildtype (=1) (Figure 2E).

### 5.3. The Ala305Val and Ala305Thr Mutations Reduce ^3^H-GABA Uptake Across HEK293T Cells, Human Astrocytes, and Human Cortical Neurons

**Figure 3:**
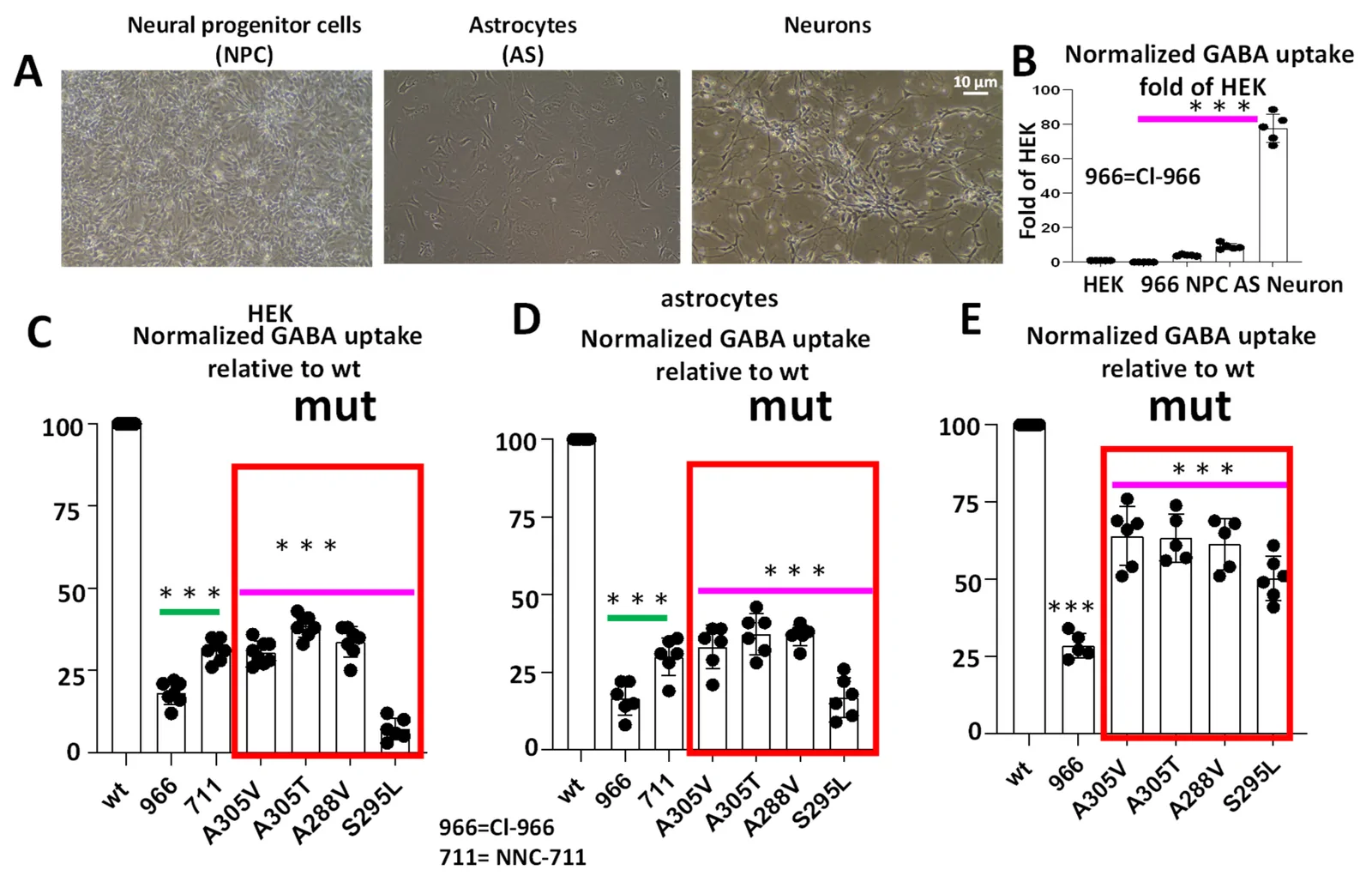
**The GABA uptake function of the mutant GAT-1 p.Ala305Val and adjacent mutations across cell types including human neurons and astrocytes.** (**A**,**B**) The live image of neural progenitor cells (NPCs), astrocytes and neurons before experiment were provided (**A**). (**B**) The graphs show the relative GABA uptake in NPCs, astrocytes and neurons normalized to the level of HEK293T cells, which is taken as 1. (**C**) HEK293T cells under passage 18 were transfected with wildtype GAT-1^YFP^ (wt), or the mutant GAT-1^YFP^ (p.Ala305Val (A305V), p.Ala305Thr (A305T), p.Ala288Val (A288V) and p.Ser295Leu (S295L) cDNAs (0.5 µg per a 35 mm^2^ dish) for 48 h before ^3^H radioactive GABA uptake assay. (**D**,**E**) Human iPSC-derived astrocytes (**D**) and cortical neurons (**E**) were transfected with the wildtype, the mutant GAT-1(A305V) or the mutant GAT-1 p.Ala305Val, GAT-1 p.Ala305Thr, GAT-1 p.Ala288Val and GAT-1 p.Ser295Leu cDNAs at day 27 after differentiation for astrocytes and 2 months after differentiation for neurons in culture. ^3^H radioactive GABA uptake assay was performed after 2 days of transfection for astrocytes and 5 days for neurons. GABA flux was measured after 30 min transport at room temperature. The influx of GABA, expressed in pmol/µg protein/min, was averaged from duplicates of two 35 mm petri dishes for each condition and for each transfection. The average counting was taken as N = 1. The untransfected condition was taken as baseline flux, which was subtracted from both the wildtype and the mutant conditions across cell types (**C**). In (**C**–**E**), the pmol/µg protein/min in the mutant was then normalized to the wildtype from each experiment, which was arbitrarily set as 100%. (In (**B**), *** *p* < 0.001 all conditions under purple line vs. HEK293T cells; in (**C**–**E**), *** *p* < 0.001 under green line vs. wt; *** *p* < 0.001 under purple line in red boxed region vs. wt, N = 5–8 different transfections). The GAT-1-selective inhibitor Cl-966 (100 µm) was applied 30 min across cell types while the GAT-3 inhibitor (SNAP5114 (30 µM)) was only applied to astrocytes (Cl/SNAP) during GABA flux. One-way analysis of variance (ANOVA) and Tukey post hoc test for multiple comparisons.

We first compared the baseline GABA uptake in HEK293T cells, the neural progenitor cells (NPCs) and the differentiated astrocytes and neurons derived from the control IPSCs (Figure 3A and Supplementary Figure S3). Consistent with our previous study [6], NPCs, astrocytes and neurons had a higher GABA uptake compared with HEK293T cells (Figure 3B). In HEK293T cells, the p.AlaA305Val variant reduced ^3^H-GABA uptake to approximately 30% of wildtype GAT-1 function, while p.Ala305Thr reduced uptake to approximately 39%, similar to the uptake in cells expressing the wildtype but treated with the GAT-1 inhibitor Cl-966 (100 μM), suggesting GAT-1 blockade (Figure 3C). In human iPSC-derived astrocytes, p.Ala305Val reduced uptake to approximately 33% of wildtype and p.Ala305Thr to approximately 37% (Figure 3D); the GAT-3-selective inhibitor SNAP5114 was used to block GAT-3 as previously described [10] and was added in each condition. In human iPSC-derived cortical inhibitory neurons, p.Ala305Val reduced uptake to approximately 64% and p.Ala305Thr to approximately 61% (Figure 3E). In all three cell types, the measurements in the mutant transporter were then normalized to the cells expressing the wildtype transporter, which was taken as 100% (wt = 100% vs. 31% for p.Ala305Val and 37% for p.Ala305Thr) (Figure 3C), human cortical astrocytes (wt = 100% vs. 33% for p.Ala305Val and 37% for p.Ala305Thr) (Figure 3D) and neurons (wt = 100% vs. 64% for p.Ala305Val and 63% for p.Ala305Thr) (Figure 3E). The GABA transport activity for all mutations was lower than the activity of wildtype GAT-1 and was comparable to the functionality of the wildtype transporter treated with GAT-1 inhibitor Cl-966 (100 µM). This is consistent across different cell types. In combination with the previous findings, this suggests reduced GABA uptake function is a major etiology underlying SLC6A1 variants. We included p.Ser295Leu mutation in the GABA uptake assay as it is the most studied mutation, and it is the representation of loss of function for SLC6A1 mutations. We also included p.Ala288Val and Ala305Thr mutations. We included p.Ala305Thr because the mutation is in the same location. We included p.Ala288Val because it is a partial loss of function mutation, which is similar to p.Ala305Val mutation.

### 5.4. GAT-1 p.Ala305Val Protein Had Increased Endoplasmic Reticulum Retention While PBA Reduced the ER-Bound GAT-1 Protein and Increased the Total GAT-1 Protein

**Figure 4:**
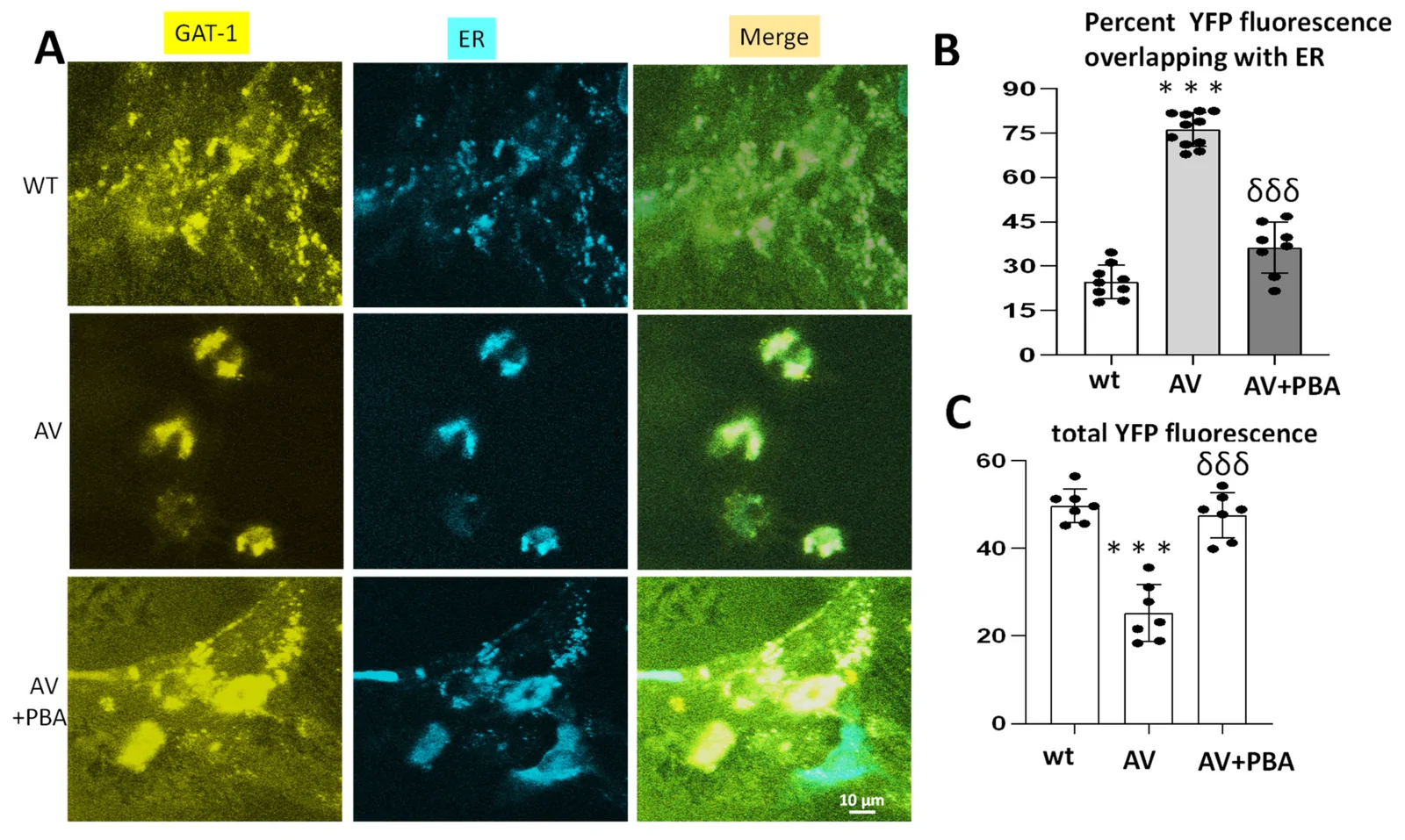
**The mutant GABA transporters GAT-1 p.Ala305Val were retained inside the endoplasmic reticulum but were relieved by 4-phenylbutyrate in the human astrocytes.** (**A**) Human astrocytes were transfected with wildtype GAT-1^YFP^ (wt) or the mutant GAT-1 p.Ala305Val (A305V, AV)^YFP^ with the pECFP-ER marker (ER^CFP^) at 1:1 ratio (0.5 µg:0.5 µg cDNAs) for 48 h. The cells were treated with vehicle or 4-phenylbutyrate (2 mM) for 24 h before confocal microscopy. Live cells were examined under a confocal microscopy with excitation at 458 nm for CFP, 514 nm for YFP. All images were single confocal sections, averaged 8 times to reduce noise, except when otherwise specified. (**B**) The GAT-1^YFP^ fluorescence overlapping with ER^CFP^ fluorescence (**B**) or the total GAT-1^YFP^ fluorescence (**C**) was quantified by Metamorph with colocalization percentage. (**C**). PBA stands for 4-phenylbutyrate (2 mM) treated for 24 h. (*** *p* < 0.001 A305V vs. wt; &&& *p* < 0.001 A305V + PBA N = 7 representative fields from 6 different transfections in 6 different batches of cells. One-way analysis of variance (ANOVA) and Newman–Keuls test was used to determine significance compared to the wt condition and between A305V and A305V/PBA. Values were expressed as mean ± S.E.M).

Live-cell confocal microscopy demonstrated that the p.Ala305Val transporter was retained intracellularly and colocalized strongly with an ER marker (ER-CFP) (Figure 4A) in human astrocytes. We chose to use human astrocytes because GAT-1 is expressed in both neurons and astrocytes and astrocytes can serve as a good tool for studying GAT-1 trafficking [10]. Approximately ~30% of wildtype GAT-1^YFP^ fluorescence overlapped with the ER marker, compared with approximately 76% for p.Ala305Val; PBA treatment reduced p.Ala305Val/ER overlap to approximately 36%, restoring an expression pattern indistinguishable from wildtype GAT-1 (Figure 4A,B). Total GAT-1 fluorescence was also lower in p.Ala305Val cells (approximately 25 vs. 49.7 arbitrary units in wildtype) and was restored by PBA to approximately 47.53 arbitrary units (Figure 4C). These results establish that the p.Ala305Val phenotype is a folding-and-trafficking deficiency rather than a substrate-binding or kinetic deficiency, and that PBA can rescue protein folding and trafficking. Together with previous findings [7,10], this suggests that increased ER retention of mutant protein and retarded wildtype protein folding due to misfolding and glycosylation arrest is a common phenomenon for mutation across GAT-1 and GABA_A_ receptor mutations.

### 5.5. Pharmacological Chaperones and ER Stress Relievers Increased the GABA Uptake Across Cell Types

**Figure 5:**
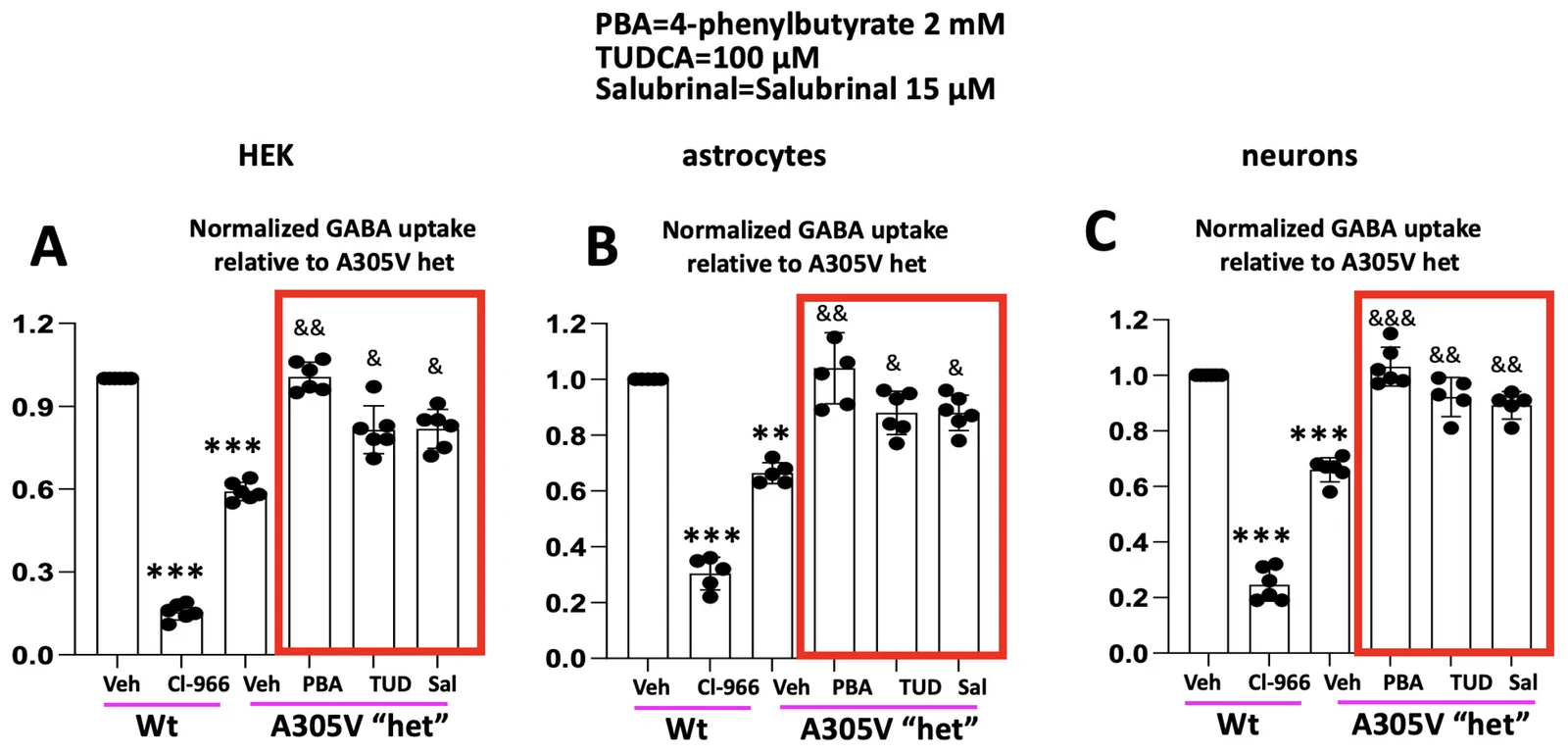
**The mutant GAT-1 Ala305Val mutant transporters were rescued by pharmacological compounds across cell types.** (**A**,**B**) HEK293T cells were transfected with wildtype GAT-1^YFP^ or with the mixture of the wildtype and the mutant GAT-1 p.Ala305Val (A305V) (0.25 µg:0.25 µg cDNAs) “heterozygously”. (**B**) The cells were then treated with vehicle (Veh), 4-phenylbutyrate (PBA, 2 mM), TUDCA (TUD, 100 µM), and salubrinal (Sal, 30 µM) for 24 h before GABA uptake experiment. In A, N = 6 batches of cells; in B, N = 5–6 batches of new cells. (**C**) Neurons: N = 5–6 batches of neurons were cotransfected with the wildtype (Wt), the mixture of the wildtype, or the GAT-1 p.Ala305Val cDNAs (0.25 µg:0.25 µg) (het). N = 5–6 batches of cells. The cells were then treated with vehicle, 4-phenylbutyrate (2 mM), TUDCA (100 µM), and salubrinal (30 µM) for 24 h before GABA uptake experiment. (In (**A**–**C**), *** *p* < 0.001; ** *p* < 0.01 vs. wt Veh; & *p* < 0.05, && *p* < 0.01; &&& vs. p.Ala305Val “Het” Veh. Two-way analysis of variance (ANOVA) and Newman–Keuls test was used to determine significance between different conditions. Values were expressed as mean ± S.E.M).

We have demonstrated that a major mechanism for PBA to rescue SLC6A1 variants is pharmacochaperoning [18]. We also demonstrated that PBA could reduce ER stress in the Gabrg2^+/Q390X^ mice of Dravet syndrome [11]. To dissect the mechanism by which PBA restores GABA uptake, the heterozygous p.Ala305Val-expressing HEK293T cells were treated with PBA, the bile-acid pharmacochaperone TUDCA and the eIF2α phosphatase inhibitor salubrinal in HEK293T cells (Figure 5A), human astrocytes (Figure 5B) and human neurons (Figure 5C). Cl-966 (50 μM) was applied as a negative control. Salubrinal slows down protein translation and prevents the accumulation of misfolded proteins, thus reducing ER stress. In HEK293T cells, the wildtype GABA uptake was taken as 1, GABA uptake of the heterozygous p.Ala305Val was 0.59 of the wildtype. The heterozygous GABA uptake increased to 1.01 with PBA; to 0.815 with TUDCA and 0.82 with salubrinal. However, compared with TUDCA and salubrinal, PBA treatment had the best efficacy. This is consistent with our previous study in a large cohort of 32 SLC6A1 variants [18]. Similarly, in both astrocytes and neurons, the GABA uptake of the heterozygous condition of p.Ala305Val was 0.66 of the wildtype; was increased to 1.04 with PBA in astrocytes and 1.03 in neurons; to 0.88 in astrocytes and 0.92 in neurons with TUDCA and 0.88 in astrocytes and 0.89 in neurons with salubrinal. Across cell types, compared with TUDCA and salubrinal, PBA treatment had the best efficacy of upregulating GABA uptake.

### 5.6. Wildtype GAT-1 DNA Augmentation Rescues SLC6A1 Mutations, and Combined Rescue Exceeds Either Intervention Alone

**Figure 6:**
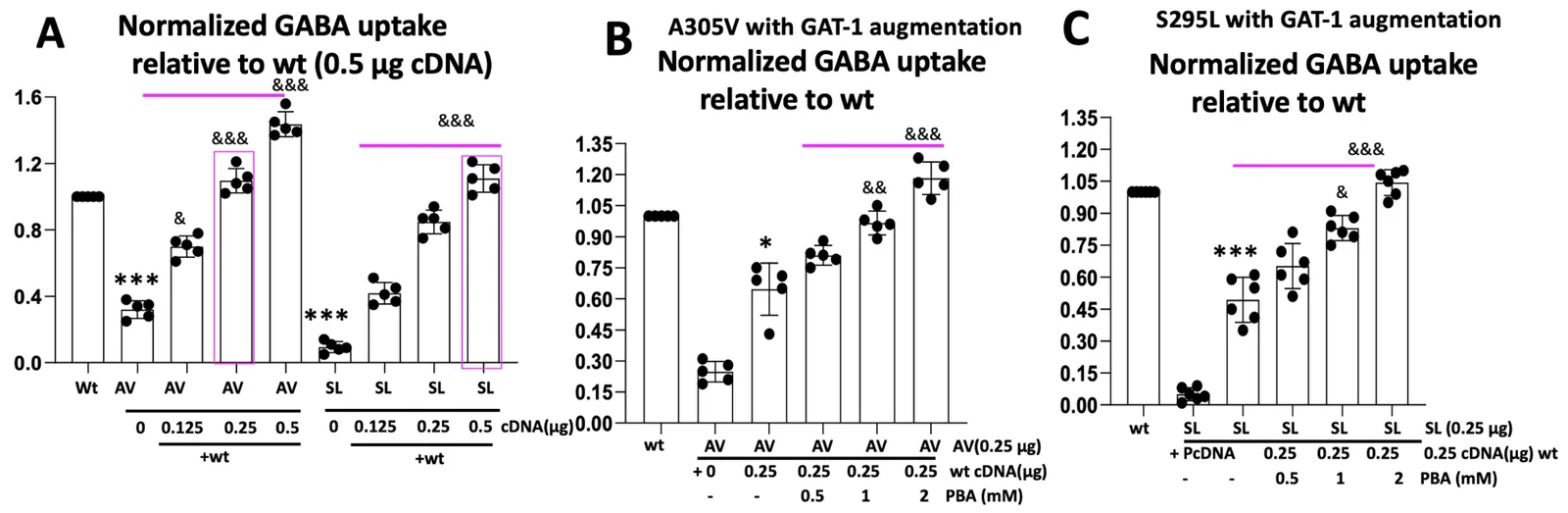
**4-phenylbutyrate reduced the dosage of wildtype allele for gene augmentation in mutant GAT-1 transporters in human astrocytes.** (**A**). Human astrocytes were transfected with wildtype (Wt) GAT-1^YFP^ (0.5 μg of GAT-1^YFP^ and 0.5 μg PcDNA for wt) or the mutant GAT-1 p.Ala305Val (A305V)^YFP^ (AV) or GAT-1 p. Ser295Leu(S295L)^YFP^ (SL) with different gene dosages of wt GAT-1^YFP^. The total DNA amount transfected was 1 μg after normalization with the vector PcDNA. (**B**,**C**). Human astrocytes were transfected with wildtype GAT-1^YFP^ or the mutant GAT-1 p.Ala305Val^YFP^ (**B**) or GAT-1 p.Ser295Leu^YFP^ (**C**) with 0.5 μg cDNA (0.25 µg of wildtype and 0.25 µg of mutant cDNA) and treated with different amounts of PBA (0, 0.5, 1 and 2 mM). In (**A**–**C**), the total cDNA amount for the wt was 0.5 μg; in the mutant conditions, the total cDNA was normalized with the vector pcDNA to the total of 0.5 μg. (In A to C, *** *p* < 0.001; * *p* < 0.01 vs. wt Veh; & *p* < 0.05, && *p* < 0.01; &&& vs. AV or SL Veh.

We here first compare the effect on GABA uptake of the mutant GAT-1 in the cells treated with PBA alone or with the wildtype cDNA upregulation at different dosages (Figure 6A–C). We then tested the GABA uptake function in the cells coexpressing the mutant GAT-1(Ala305Val) (AV, 0.25 μg) or GAT-1 p.Ser295Leu (SL, 0.25 μg) with different cDNA amounts of the wildtype allele (Figure 6A). Augmentation of the wildtype GAT-1 cDNA dose-dependently increased the GABA uptake (0.70 for 0.125 μg, 1.1 for 0.25 μg and 1.4 for 0.5 μg for p.Ala305Val; 0.418 for 0.125 μg, 0.848 for 0.25 μg and 1.11 for 0.5 μg for p.Ser295Leu vs. wt = 1). We then tested the GABA uptake function in the cells coexpressing the equal amounts of mutant GAT-1 and the wildtype GAT-1 cDNAs and then treated with different concentrations of PBA (0.5 to 4mM) (Figure 6B,C). Cell viability was compromised in the dishes treated with 4 mM PBA, which thus was not included. In both p.Ala305Val (0.65 for veh; 0.81 for PBA 0.5 mM; 0.966 for PBA 1 mM and 1.18 for PBA 2 mM) and p.Ser295Leu (0.49 for veh; 0.65 for PBA 0.5 mM; 0.83 for PBA 1 mM and 1.04 for PBA 2 mM) mutation conditions, PBA (2 mM) increased the GABA uptake and reached the maximal GABA uptake functionality. This suggests that the combined approach of PBA plus the wildtype gene augmentation is more favorable than either approach alone. An optimal gene function restoration can be achieved with a lower dose of gene therapy plus PBA treatment.

**Figure 7:**
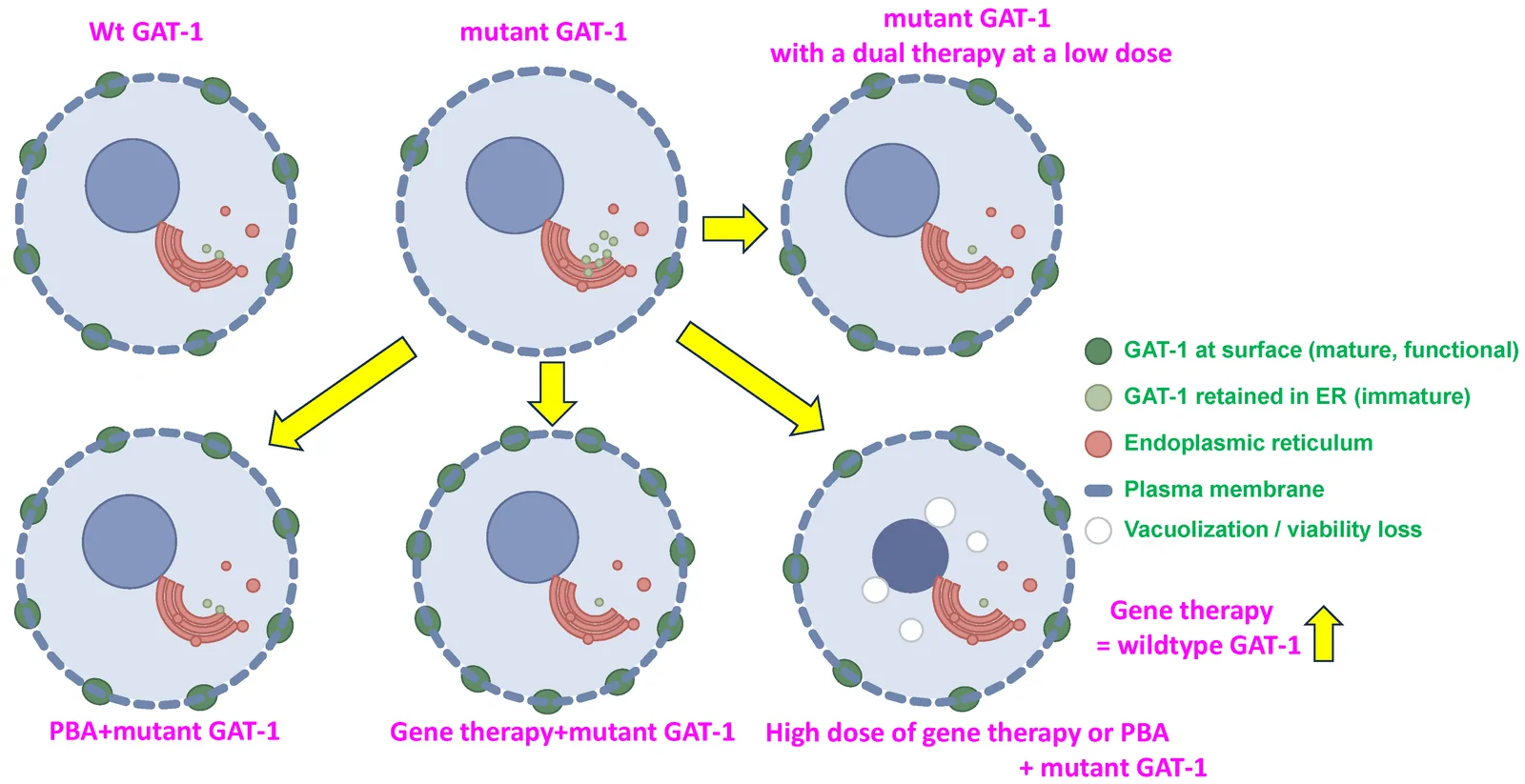
**A schematic presentation of the low dose of 4-phenylbutyrate can upregulate the functional copy of wildtype GAT-1 from gene therapy.** We propose that 4-phenylbutyrate (PBA) facilitates the folding and trafficking of wild-type GAT-1 while promoting the clearance of unfolded or misfolded mutant proteins, thereby maximizing functional GAT-1 expression at the cell membrane without inducing endoplasmic reticulum stress or cellular toxicity. By restoring cellular proteostasis and improving the processing of newly synthesized GAT-1, PBA may also enhance the therapeutic efficacy of emerging gene replacement and antisense oligonucleotide (ASO)-based therapies, making it an attractive adjunctive treatment for precision medicine approaches.

Based on the findings from above, we propose that 4-phenylbutyrate restores proteostasis by promoting the folding, maturation, and trafficking of wild-type GAT-1 while facilitating the clearance of unfolded or misfolded protein species, thereby maximizing functional GABA transporter 1 expression at the plasma membrane without inducing endoplasmic reticulum stress or cellular toxicity. We further hypothesize that optimizing the intracellular protein-folding environment will improve the expression and functional maturation of GAT-1 produced by gene replacement therapies and increase the effectiveness of antisense oligonucleotide (ASO)-based strategies, positioning 4-phenylbutyrate as a complementary therapeutic platform for current and next-generation precision therapies (Figure 7).

## 6. Discussion

This study identifies SLC6A1 p.Ala305Val as a trafficking-impaired, loss-of-function GAT-1 variant whose dysfunction can be rescued by pharmacologic and genetic interventions acting on distinct but convergent steps in GAT-1 transporter biogenesis. There are four main findings in this study supporting this notion. First, computational stability predictors uniformly indicate destabilization at p.Ala305 for both p.Ala305Val and the residue-matched p.Ala305Thr comparator, with a magnitude consistent with disruption of transmembrane packing. Second, ^3^H-GABA uptake is reduced across HEK293T cells, human iPSC–derived astrocytes, and cortical neurons expressing Ala305Val to a degree similar to the cells expressing the wildtype but treated with GAT-1 specific pharmacological inhibitors such as Cl-966 and NNC-711. Third, the mutant GAT-1 accumulates in the ER and the total mature GAT-1 pool is reduced, suggesting the lesion at folding and forward trafficking rather than at substrate binding or surface pharmacology. Fourth, the deficit is reversible: PBA, TUDCA, salubrinal, in addition to our previous studies on BiP and calnexin overexpression, BIX-mediated BiP activation [18] and wildtype GAT-1 cDNA augmentation each independently improve GABA uptake function, and the combination of PBA with GAT-1 gene augmentation produces rescue greater than either single intervention in the available experiments.

The mechanistic implication is that SLC6A1-related DEEs, at least for trafficking-impaired missense variants such as p.Ala305Val and p.Ser295Leu, are not pure dose-haploinsufficiency diseases of the affected gene and not pure pharmacochaperone-responsive misfolding diseases. They are both. Adding more wildtype GAT-1 helps because the absolute pool of the transporter is rate-limiting. Adding PBA helps because the fraction of that pool that successfully folds and traffics is rate-limiting. The two strategies therefore act on different segments of the same biogenesis pathway and should be designed to cooperate in a future precision-medicine framework. This framing predicts that pharmacochaperone responsiveness will vary across SLC6A1 variants, with the largest benefit from gene augmentation plus PBA.

The clinical relevance of these findings is direct and the translatability is high. PBA and its commercial form glycerol phenylbutyrate (Ravicti) are already in clinical use for unrelated indications, and an investigator-initiated trial in SLC6A1- and STXBP1-related DEEs is in progress (NCT04937062). The data presented here support continued evaluation of PBA therapeutics in SLC6A1-related disease, and indicate that variant-level functional and trafficking characterization is necessary to guide precision deployment. The data also justifies continued preclinical development of GAT-1 gene-augmentation strategies in parallel with chemical chaperoning, with the expectation that the two modalities will be most effective in combination and can de-risk the potential overdose of gene therapy.

Several limitations of the study should be acknowledged. Although three independent cell systems were interrogated, in vivo confirmation of combined PBA-plus-wildtype GAT-1 augmentation rescue in an p.Ala305Val knock-in mouse model is necessary to establish translational validity. The wildtype allele augmentation experiments use plasmid co-expression as a model of gene therapy, which allows us to evaluate the effect of PBA in addition to gene therapy and PBA without the variables and tropism from viral or non-viral delivery in vivo. Finally, the analyses here address only a few mutations at adjacent residues; generalization to the broader SLC6A1 variant landscape will require systematic characterization of pharmacochaperone responsiveness across a representative variant panel, ideally coupled to in silico stability prediction that has been calibrated against functional readouts.

Despite these limitations, the central conclusion of this study is robust: SLC6A1 p.Ala305Val causes a well-defined trafficking-linked transporter deficit; that deficit is reversible by interventions acting on protein folding and on transporter gene dose, and the combination provides a rational and translationally accessible framework for precision therapy in SLC6A1-related DEEs. PBA can enhance the folding of functional copy delivered from gene therapy by maximally leveraging the existing foldable copies of GAT-1. Importantly, the rescue experiments were performed under heterozygous conditions, which more closely recapitulates the patient’s genotype. In this context, the mutant protein may exert a dominant-negative effect by aberrantly oligomerizing with the wildtype protein or by impairing wild-type protein folding through sequestration or excessive engagement of molecular chaperones. However, PBA can enhance the chaperone expression and remove the unfolded or misfolded protein more efficiently. Thus, it is likely that no or little dominant negative effect will exist in the cells expressing the mutant GAT-1 p.Ala305Val treated with PBA.

## 7. Conclusions

SLC6A1 p.Ala305Val is a de novo missense variant that destabilizes GAT-1, increases endoplasmic reticulum retention, and reduces GABA uptake across heterologous cells and human iPSC-derived astrocytes and cortical neurons. Pharmacologic chaperones (PBA, TUDCA), ER-stress modulators (salubrinal), and genetic supplementation of the wildtype allele each independently rescue GAT-1 GABA uptake function, and the combination of PBA with gene augmentation produces rescue greater than either alone. These findings define a coherent two-axis therapeutic approach: folding correction plus wildtype GAT-1-dose augmentation for SLC6A1-related DEEs, and motivate variant-level characterization as the next step toward precision medicine.

## Data Availability

This study does not involve data reporting. Any raw data for functional assays can be made available upon request. Any clinical information can be made available upon request, subject to approval by the appropriate ethical board.

## Supplementary Materials

The following supporting information can be downloaded at: https://www.mdpi.com/article/10.3390/genes17080983/s1, Figure S1:Delta Delta G values of Ala305Thr mutation; Figure S2: Full-length gel of Figure 2C; Figure S3: Live images of human neural pregenital cells, astrocytes and neurons before experiment.
