# Supplementary material for: 4-Phenylbutyrate Plus Wildtype GAT-1 Augmentation: A Dual Therapy to Rescue SLC6A1 Variant-Associated Developmental and Epileptic Encephalopathy
Source: Genes (Basel). 2026 Aug 21;17(8):983. doi: 10.3390/genes17080983 (PMC13512211; doi:10.3390/genes17080983)
Supplement: Supplementary file 1 [file genes-17-00983-s001.zip › genes-4381080-supplementary.pdf]

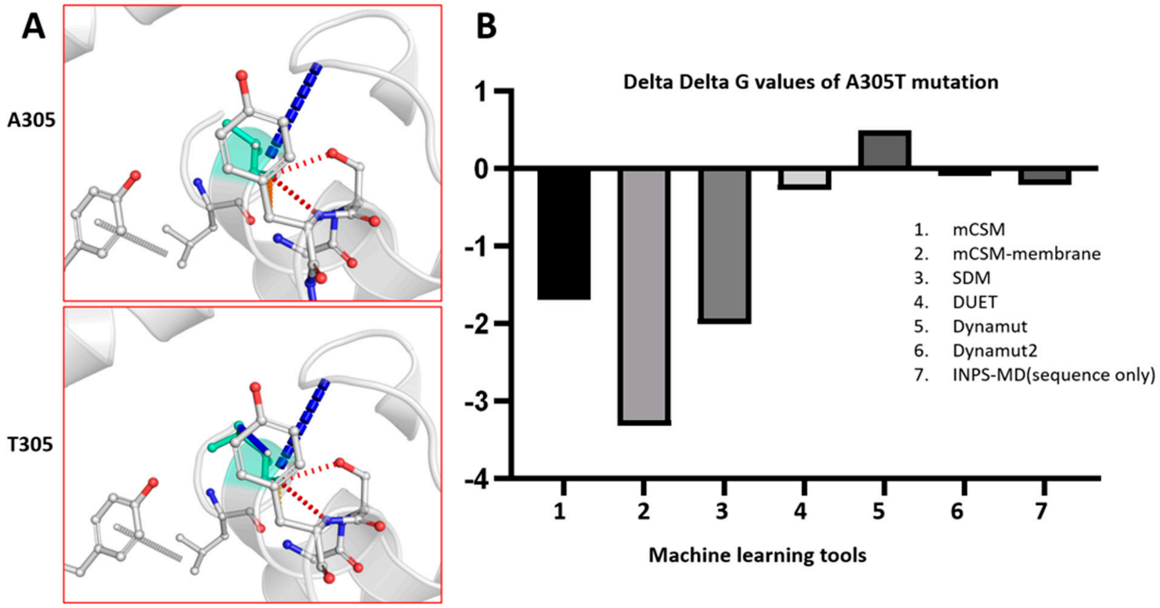

Figure S1. Delta Delta G values of Ala305Thr mutation.

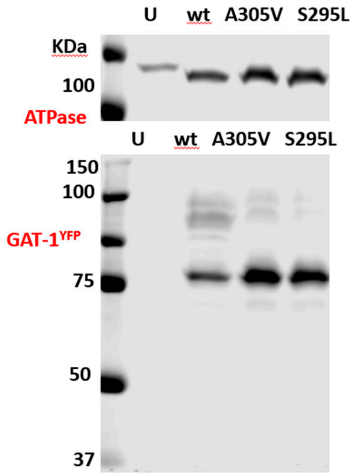

Figure S2. Full-length gel of Figure 2C.

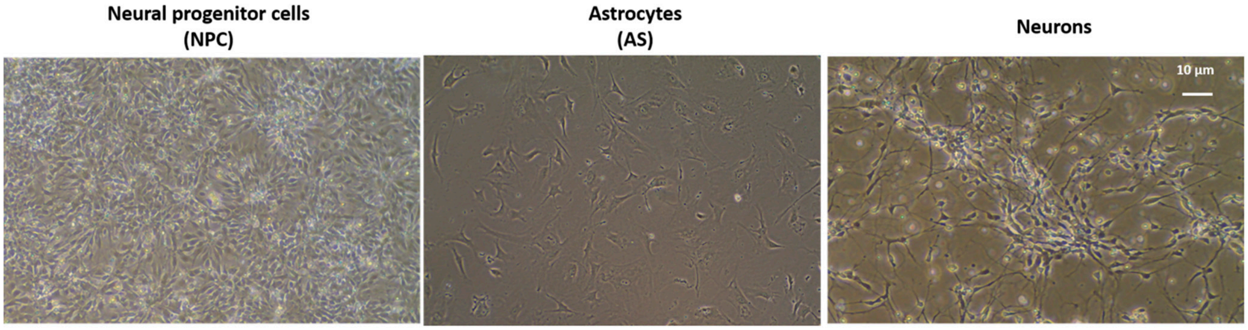

Figure S3. Live images of human neural pregenital cells, astrocytes and neurons before experiment.
